# Endovascular Repair of 100 Urgent and Emergent Free or Contained Thoracoabdominal Aortic Aneurysms Ruptures. An International Multicenter Trans-Atlantic Experience

**DOI:** 10.1097/SLA.0000000000006231

**Published:** 2024-02-07

**Authors:** Paolo Spath, Nikolaos Tsilimparis, Enrico Gallitto, Daniel Becker, Andrea Vacirca, Bärbel Berekoven, Giuseppe Panuccio, Angelos Karelis, Andrea Kahlberg, Germano Melissano, Nuno Dias, Tilo Kölbel, Martin Austermann, Gianluca Faggioli, Gustavo Oderich, Mauro Gargiulo

**Affiliations:** * Vascular Surgery, Department of Medical and Surgical Sciences (DIMEC), University of Bologna, Bologna, Italy; † Department of Vascular Surgery, Ludwig-Maximillian University Hospital, Munich, Germany; ‡ Department of Vascular Surgery, Hospital “Infermi” Rimini, AUSL Romagna, Rimini, Italy; § IRCCS University Hospital Policlinico S. Orsola Bologna, Italy; ∥ Department of Vascular Surgery, Inselspital, University Hospital Bern, Bern, Switzerland; ¶ Department of Cardiothoracic and Vascular Surgery, University of Texas, Houston, TX; # Clinic for Vascular Surgery, St. Franziskus Hospital GmbH, Münster, Germany; ** German Aortic Center, Department of Vascular Medicine, University Medical Center Eppendorf, Hamburg, Germany; †† Department of Clinical Sciences, Vascular Center Malmö, Skåne University Hospital, Lund University, Malmö, Sweden; ‡‡ Department of Vascular Surgery, Vita-Salute University, San Raffaele Scientific Institute, Milan, Italy

**Keywords:** endovascular repair, major adverse events, mortality, pulmonary complications, ruptured thoracoabdominal aortic aneurysm, survival, technical success

## Abstract

**Objective:**

To analyze the outcomes of urgent/emergent endovascular aortic repair of patients with free/contained ruptured thoracoabdominal aortic aneurysms (rTAAA).

**Background:**

Endovascular repair of rTAAA has been scarcely described in emergent setting.

**Methods:**

An international multicenter retrospective observational study (ClinicalTrials.govID:NCT05956873) from January 2015 to January 2023 in 6 European and 1 US Vascular Surgery Centers. Primary end points were technical success, 30-day and/or in-hospital mortality, and follow-up survival.

**Results:**

A total of 100 rTAAA patients were included (75 male; mean age 73 years). All patients (86 contained and 14 free ruptures) were symptomatic and treated within 24 hours from diagnosis: multibranched off-the-shelf devices (Zenith t-branch, Cook Medical Inc., Bjaeverskov, Denmark) in 88 patients, physician-modified endografts in 8, patient-specific device or parallel grafts in 2 patients each. Primary technical success was achieved in 89 patients, and 30-day and/or in-hospital mortality was 24%. Major adverse events occurred in 34% of patients (permanent dialysis and paraplegia in 4 and 8 patients, respectively). No statistical differences were detected in mortality rates between free and contained ruptured patients (43% vs 21%; *P*=0.075). Multivariate analysis revealed contained rupture favoring technical success [odds ratio (OR): 10.1; 95% CI: 3.0–33.6; *P*<0.001]. Major adverse events (OR: 9.4; 95% CI: 2.8–30.5; *P*<0.001) and pulmonary complications (OR: 11.3; 95% CI: 3.0–41.5; *P*<0.001) were independent risk factors for 30-day and/or in-hospital mortality. The median follow-up time was 13 months (interquartile range 5–24); 1-year survival rate was 65%. Aneurysm diameter >80 mm (hazard ratio: 2.0; 95% CI: 1.0–30.5; *P*=0.037), technical failure (hazard ratio: 2.6; 95% CI: 1.1–6.5; *P*=0.045) and pulmonary complications (hazard ratio: 3.0; 95% CI: 1.2–7.9; *P*=0.021) were independent risk factors for follow-up mortality.

**Conclusions:**

Endovascular repair of rTAAA shows high technical success; the presence of free rupture alone appear not to correlate with early mortality. Effective prevention/management of postoperative complications is crucial for survival.

Thoracoabdominal aortic aneurysm (TAAA) is the most extensive form of aortic pathology.^1,2^ Traditional open surgical repair has been associated with high perioperative mortality/morbidity, although specialized centers have made significant progress.^2–4^ In recent years, fenestrated and branched endovascular aneurysm repair (FB-EVAR) has emerged as an effective treatment option for patients with complex abdominal aortic aneurysms^5–9^ who have suitable anatomy. Early and mid-term results in elective patients treated for TAAAs compare favorably with historical results of open repair.^8–12^

Ruptured TAAA (rTAAA) represents a formidable challenge requiring immediate treatment.^1^ Despite these challenges, in the last aortic guidelines of the American Heart Association, open surgical repair was recommended for patients with hemodynamic instability, with endovascular repair reserved for patients with stable ruptures in centers with access to these devices and expertise. However, despite these recommendations, open surgical repair carries high mortality and morbidity in the setting of aortic rupture.^1,2,13^ The increasing availability of off-the-shelf multibranched stent grafts has expanded the indications of endovascular approaches, avoiding the 6 to 8 weeks time delay for patient-specific devices.^11,14–17^ In centers that do not have access to off-the-shelf devices or in patients without anatomic requirements, physician-modified endografts (PMEGs),^18^ in situ fenestrations or parallel grafts (PGs) may be used.^19,20^ Several small single and multicenter experiences have demonstrated promising results for treatment of urgent and symptomatic unruptured TAAAs, but there is the paucity of data on this indication.^21–24^ A large multicenter study showed favorable results nonelective cases, but this study also included symptomatic intact aneurysms treated emergently.^25^ There is only a small series that described the results of FB-EVAR for rTAAA with early mortality of 27%.^26^ Aside from the recent American Heart Association guidelines, there is not a position statement of international vascular and endovascular surgical societies on the role of endovascular repair for rTAAAs.^27–29^ The aim of this study is to analyze the outcomes of endovascular aortic repair using fenestrated/branched endografts (off-the-shelf or customized), PMEGs, and parallel grafts (PGs) for both free and contained rTAAAs.

## METHODS

### Study Design and Patient Selection

This was an international multicenter retrospective observational cohort study (ClinicalTrials.gov ID: NCT05956873) to assess the technical success, early and late patient survival outcomes of endovascular aortic repair in patients with free ruptured and contained rTAAA.

### Inclusion and Exclusion Criteria

**FIGURE 1:**
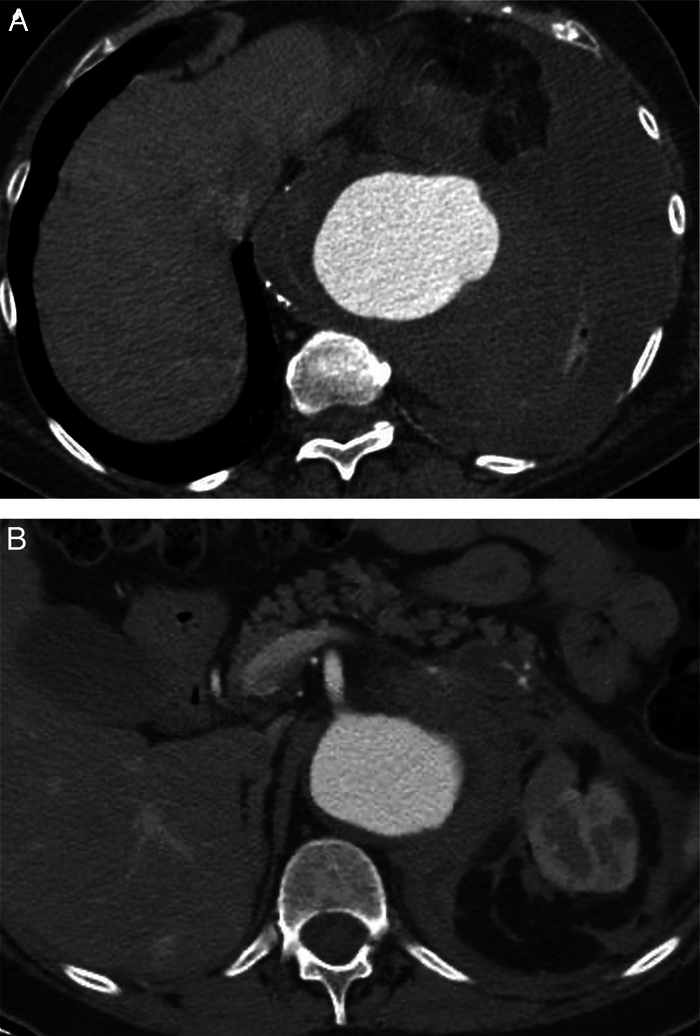
Radiological differences between free and contained rupture. A, Free rupture at the level of the thoracic aorta, with evidence of bleeding outside the aortic wall and in this specific case with left haemothorax and complete lung atelectasis. B, Contained rupture at the level of the renal and mesenteric arteries in a IV type rTAAA, with total loss of the integrity of the suprarenal right aortic wall, without clear evidence of bleeding but with periaortic structures hematoma.

The study included adult patients diagnosed with free or contained rTAAAs, including Crawford Extent I to V TAAAs.^30^ Patients with intact asymptomatic or symptomatic TAAAs were excluded. Rupture status was confirmed preoperatively by the review of computed tomography angiography. Patients treated for recurrent or enlarging aneurysms after previous open or endovascular aortic repair, saccular aneurysms, penetrating aortic ulcers, and chronic postdissection aneurysms were also included as long as the proximal landing zone was based on the supra-celiac aorta consistent with at least an Extent IV TAAA repair.^31^ A free rupture was defined as the presence of aortic rupture with evidence of intraperitoneal or pleural hemorrhage (Fig. 1A). A contained rupture was defined by lack of integrity of the aortic wall with associated periaortic hematoma, but no evidence of intracavitary hemorrhage (Fig. 1B).^27,29,30,32^ Patients were also considered with respect to hemodynamic stability, with unstable rTAAA defined as cardiopulmonary arrest or inability to maintain systolic blood pressure >90 mm Hg despite intravenous fluid and vasopressor support.^30^

**FIGURE 2:**
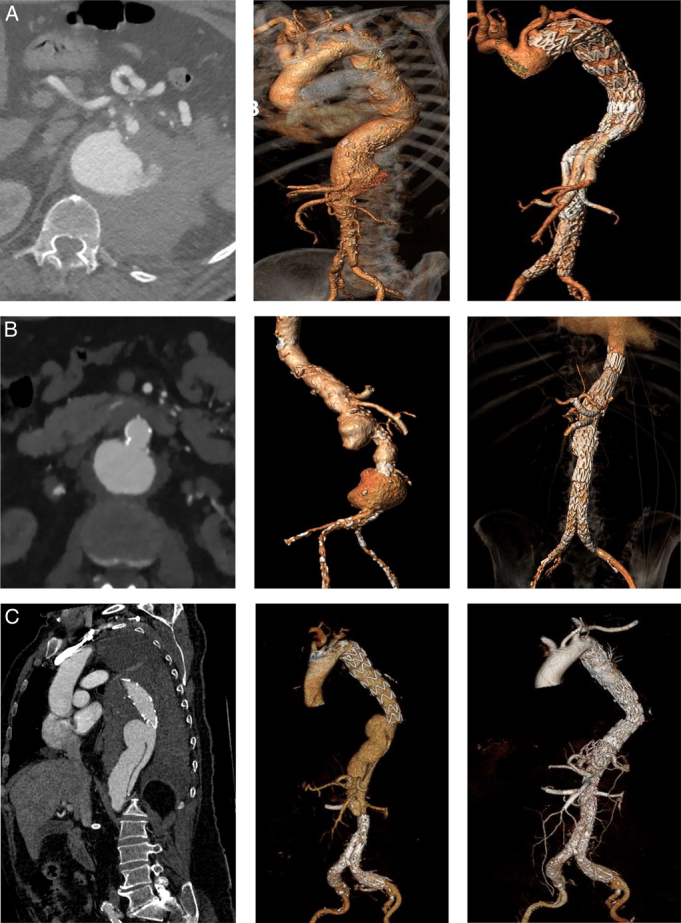
Examples of preoperative computed tomography angiography (CTA), preoperative 3-dimensional volume rendering (3D VR) and postoperative 3D VR of ruptured thoracoabdominal aortic aneurysms (rTAAA) cases repaired using off-the-shelf (A), custom-made (B), and physician-modified endograft (C) techniques. A, Case of contained rupture type II rTAAA treated with 2 proximal thoracic endografts [thoracic endovascular repair (TEVAR)], and with the use of an off-the-shelf multibranched Cook t-branch and a bifurcated abdominal graft. B, Case of a double contained rupture at the level of the sura-visceral and visceral aorta and infrarenal aorta, treated with the use of a proximal TEVAR, a custom-made device of a dead patient with two proximal branches for celiac trunk and superior mesenteric artery and 2 fenestrations for renal arteries. The patient was on 3 times week dialysis and the 2 fenestrations were occluded with an aortic cuff. The cases was completed with a distal bifurcated graft. C, Case of a free aortic rupture of a postdissection TAAA below a previous implanted TEVAR and previous distal endovascular abdominal repair (EVAR), treated with the use of a thoracic stent-graft with 4 physician-modified fenestrations for celiac trunk, superior mesenteric artery, and renal arteries.

All patients underwent emergent or urgent endovascular repair within the first 24 hours of rupture diagnosis made by urgent computed tomography angiography. Device options included off-the-shelf multibranched stent grafts, patient-specific fenestrated-branched devices, physician-modified/in-situ fenestrations endografts with fenestrations and/or directional branches and PGs for renal and visceral arteries (Fig. 2).

### Primary End Point Definition

The primary procedural endpoint was technical success, defined accordingly to reporting standards.^30^ Primary clinical endpoint was 30-day and/or in-hospital mortality, which was defined as any mortality occurring during the operative procedure, within the first 30 days or during the hospital stay if hospitalization period was longer than 30 days. The primary end point during the follow-up period was 1-year overall patient survival, regardless aortic pathology is defined as the cause of death.

### Secondary End Points Definitions

Secondary procedural end points included type of endovascular repair, proximal and distal landing zone, adjunctive procedures, procedure duration, iodinated contrast volume, any immediate, 30-day, and follow-up endoleaks requiring interventions. Secondary clinical end points at 30 days/discharge time were major adverse events (MAEs) and clinical success, as described by reporting standards,^30^ 90-day mortality, and any procedure-related reinterventions. Secondary follow-up end points were cumulative incidence of aortic-related death beyond 90 days and any type of reintervention related to the index procedure.

### Subgroup Analysis

Subgroup analysis examined preoperative, intraoperative, and postoperative differences between patients presenting with free and contained aortic ruptures.

### Risk Factors Analysis

Risk factors were analyzed using univariate analysis, and independent factors were determined through multivariate analysis for the study’s primary end points (technical success, 30-day and/or in-hospital mortality, follow-up survival). The presence of an aneurysm diameter >80 mm was reported as a preoperative risk factor based on the increased risk of aortic events.^33^

### Participating Centers

Six European and 1 center in the United States accepted to participate. Ethical approval was obtained from the principal investigator’s hospital local board (LMU 22-0483), and individual centers obtained approval according to their local guidelines for retrospective anonymous data collection.

### Regulatory Approval and Statistical Analysis

Analysis adhered to the standardized reporting standards for endovascular aortic repair set by the Society for Vascular Surgery/American Association for Vascular Surgery^30^ and followed “Strengthening the Reporting of Observational Studies in Epidemiology” guidelines for data assessment.^34^ Statistical analysis used SPSS version 28.0 (IBM Corp, Armonk, NY). Aiming over 50 patients for follow-up analysis, the intended sample size was 75 patients, accounting for 10% missing data and rough early mortality of 20% to 30%,^21,23,24,26^ minimum target was 83 patients.

Categorical variables were analyzed using Pearson χ^2^ test and quantitative variables were analyzed using parametric/nonparametric tests. A *P* value below 0.05 was considered significant. After univariate analysis, multivariate analysis on significant factors was performed to adjust for confounders. Time-to-event outcomes were analyzed using Kaplan-Meier curves and Log-rank test, and Cox regression analysis was used for multivariate analysis.

## RESULTS

### Patient Selection and Clinical Data

A total of 100 patients were treated for rTAAAs, including 75 male (75%) patients and mean age of 73±7 years old (Table 1). Aneurysm extent was Crawford I to III in 43 patients and Crawford IV in 57 patients. All patients presented with thoracic and/or abdominal pain and had imaging confirmation of contained rTAAA in 86 patients or free rTAAA in 14 patients (Supplementary Table S1, Supplemental Digital Content 1, http://links.lww.com/SLA/F13). Hemodynamic instability at the time of presentation occurred in 25 patients (free ruptures 5/14–36% vs contained ruptures 20/86–23%). The mean time from diagnosis to treatment was 12±9 hours (Table 1) aiming to accurately plan and stabilize the patient whenever possible, despite the expedited procedure time-frame.

**TABLE 1:** Baseline Characteristics and Clinical Presentation of Included Patients

Variable	Number or mean (SD)		Number or mean (SD)
Overall cases	100 (100)	Overall cases	100 (100%)
Preoperative characteristics		Clinical Presentation	
Age, years	73 (7)	Mean systolic pressure	122 (35)
Male	75	Mean diastolic pressure	75 (12)
Coronary artery disease	35	Ammine support	25
Peripheral arterial disease	21	Time diagnosis-treatment (h)	12 (9)
COPD	25	Treatment within first 3 hours	28
Chronic kidney disease	35	Rupture details	
Dialysis	4	Symptoms	100
Hypertension	90	Abdominal pain	39
Diabetes mellitus	11	Chest pain	7
Smoking history	66	Back/lumbar pain	21
Obesity	36	Syncope	33
Dyslipidemia	48	Location of rupture (SVS zones)	
Previous stroke/TIA	13	Zone 3	3
Previous aortic repair (open surgery)	19	Zone 4	9
Previous aortic repair (endovascular)	22	Zone 5	27
ASA score (III–IV)*	95	Zone 6	9
Clinical classification		Zone 7	4
Mean aortic diameter (mm)	76 (8)	Zone 8	9
Crawford extension I-III	43	Zone 9	39
Type I	4	Signs of hematoma/active bleeding	41
Type II	23	Need for hematoma evacuation	8
Type III	16	Thoracotomy	5
Suprarenal/Crawford extension IV	57	Laparotomy	3

Data are presented as median (interquartile range-IQR) and categorical data as numbers (percentage).

*ASA indicates American Society of Anesthesiologists; COPD, chronic obstructive pulmonary disease.

### Procedural Details and Intraoperative Results

Device selection was off-the-shelf multibranch stent graft in 88 patients (Zenith t-branch, Cook Medical, Bloomington, IN), fenestrated PMEGs in 8 and patient-specific or PGs in 2 patients each. The type of repair in relation to patient presentation and procedural details are summarized in Table 2. All patients who presented with free rTAAAs were treated using off-the-shelf devices (Supplementary Table S1, Supplemental Digital Content 1, http://links.lww.com/SLA/F13), except for one patient who received a PMEG. PMEGs were performed in 4 of the 7 participating centers. The 2 patients treated by patient-specific devices had either a previously designed stent graft or a device adapted from a deceased patient.

**TABLE 2:** Type of Endovascular Repair

Type of repair	Patient, N	Free rupture N (%)	Contain rupture N (%)	TVV targeted /target	TVV lost/occluded/pre-op absence	Upper limb access, N (%)	Femoral percutaneous, N (%)	Proximal TEVAR, N (%)	Distal tube graft, N (%)	Distal bifurcated Graft, N (%)	IBD, N (%)	TS, N (%)
T-branch	88	13 (15)	75 (85)	341/350	3/6/2	77 (88)	42 (48)	51 (58)	29 (33)	59 (67)	16 (18)	77 (88)
CMD	2	0	2 (100)	6/8	0/2/0	1 (50)	0	0	1 (50)	1 (50)	0	2 (100)
PMEG	8	1 (13)	7 (87)	24/24	0/0 /8	4 (50)	8 (100)	3 (39)	6 (75)	2 (25)	0	8 (100)
Parallel grafts	2	0	2 (100)	6/6	0/0/2	2 (100)	2 (100)	1 (50)	2 (100)	0	0	2 (100)
OVERALL	**100**	**14**	**86**	**377**/**388**	**3**/**8/12**	**84**	**52**	**55**	**38**	**62**	**16**	**89**

Bold represent the overall number of each item.

Data are presented as numbers (percentage).

CMD indicates custom-made devices; IBD, iliac branch devices; TS, technical success; TVV, target visceral vessels.

A total of 74 adjunctive procedures were performed in 63 patients to optimize access or sealing zones (Supplementary Table S2, Supplemental Digital Content 1, http://links.lww.com/SLA/F13). The mean procedure time was 354±187 minutes and was significantly longer among patients with free as compared with contained ruptures (446±310 vs 339±212 minutes, *P*=0.006). The mean iodinated contrast volume was 221±103 mL, with no difference between free and contained ruptured cases. Prophylactic spinal fluid drainage was performed in 30 patients, all of whom had contained ruptures and hemodynamic stability. Systemic heparinization was individualized on patient’s condition: about 200 seconds of activated clotting time for contained ruptures, based on coagulation status for free ones. Five patients required thoracotomy for evacuation of intrapleural hematoma and permit pulmonary re-expansion (3 immediate chest tube); 3 patients had abdominal cavity decompression to facilitate ventilation and bowel perfusions. Procedures were performed after multidisciplinary evaluation with thoracic and general surgeons, weighing the risks of recurrent bleeding after decompression and the need to stabilize the pulmonary/bowel status.

### Primary End Points

Primary technical success was achieved in 89 patients, including 2 intraoperative mortalities. Details of technical failure and solutions are specified in Supplementary Table S3, Supplemental Digital Content 1, http://links.lww.com/SLA/F13, achieving an overall assisted technical success rate of 93% after well solving 4 endoleaks.

There were 24 mortalities within 30 days and/or hospital stay. In addition to 2 intraoperative mortalities, 2 patients died within the first 48 hours, and 3 patients died beyond 30 days during the index hospitalization. The median overall length of stay was 13 days (IQR=8–21). Main causes of mortality were multisystem organ failure in 10 patients, followed by respiratory, cardiac, and hemorrhagic complications in 7, 4, and 3 patients, respectively. No preoperative risk factors, such as type or rupture (contained vs free), location of rupture (abdominal vs thoracic), extension of aneurysm (Extent I-III vs IV TAAA), or hemodynamic status/vasopressor support resulted in significant factors (Table 3). The median follow-up time was 13 months (IQR=5–24) and the 1-year patient survival rate was 65% (SE 5%) (Supplementary Figure S1, Supplemental Digital Content 1, http://links.lww.com/SLA/F13).

**TABLE 3:** Significative Risk Factors Associated With 30-day+ In-hospital Mortality

Risk factor (RF)	Overall events N/total patients (%)	N deaths/overall patients with RF (%)*	N deaths/overall patients without RF (%)*	*P* †
Intraoperative	Total N=100	—	—	—
Technical failure	11 (11%)	7/11 (63)	17/89 (19)	0.004
Postoperative	Total N=98	—	—	—
ICU>48 h	69 (70)	20/69 (29)	2/29 (7)	0.017
MAEs	33 (34)	17/33 (51)	5/65 (8)	<0.001
Pulmonary complications	19 (19)	13/19 (68)	9/79 (12)	<0.001
Acute kidney injury	22 (22)	9/22 (41)	13/76 (17)	0.039
Spinal cord ischemia	20 (20)	8/20 (40)	14/78 (18)	0.025
Bowel ischemia	5 (5)	4/5 (80)	18/93 (19)	0.002
30-d reinterventions	24 (24)	9/24 (38)	13/74 (18)	0.042

Data of exclusive statistical significant data are reported in the table together with the *P* value level (significant if <0.05).

* Overall 30-day+in-hospital mortality patients 24: 2 intra-procedural deaths, 3 deaths after 30 days Data are presented as fractions over total patients and (percentage).

† *P* value based on χ^2^ test.

ICU indicates intensive care unit; RF, risk factor.

### Secondary End Points

The main postoperative primary and secondary end points are presented in Supplementary Table 4, Supplemental Digital Content 1, http://links.lww.com/SLA/F13. A total of 33 patients (34%) experienced MAEs, with 22 cases having more than 1 concomitant MAE. Patients with renal injuries needing dialysis were 12; among them, 4 patients were permanent. Among the 20 SCI cases, 12 were transient with full recovery before discharge, while 8 patients had permanent lesions, including 4 cases of bilateral paralysis that were diagnosed on the first postoperative day. Postoperative reinterventions were necessary for 24 patients (Supplementary Table S5, Supplemental Digital Content 1, http://links.lww.com/SLA/F13) due to endoleak in 7 cases that needed immediate and prompt treatment to avoid the risk of subsequent bleeding. Clinical success at patient’s discharge was reported in 68 patients. Overall, a total of 27 patients died within the first 90 days.

Among follow-up mortalities (n=24), 2 contained ruptured treated patients experienced aortic-related death after rupture after 6 and 20 months, respectively, due to the persistence of endoleaks. The cumulative aortic-related death after the first 90 days was reported as 28% at 1 year. The other mortalities were not considered aortic related.

During follow-up, freedom from reintervention rate was 83% at 1 year (Supplementary Figure S2, Supplemental Digital Content 1, http://links.lww.com/SLA/F13). Among the 15 reported reinterventions, target visceral vessels–related endoleaks occurred in 9 cases, while main endograft-related endoleaks were reported in 5 cases. One original type IV TAAA case, primarily treated with a t-branch for contained rupture, experienced aneurysm rupture after 4 years and survived after open conversion.

### Free Versus Contained Rupture Analysis

Subgroup analysis is reported in Table 4. Free rupture cases had shorter time from diagnosis to treatment (*P*=0.009) with a lower primary technical success rate (8/14, 57% vs 81/86, 94%, *P*<0.001). In the postoperative period, free ruptured patients had a higher rate of MAEs (69% vs 28%, *P*<0.001), pulmonary complications (77% vs 11%, *P*<0.001), and early reinterventions (62% vs 19%, *P*<0.001). No differences were detected in intraoperative and postoperative mortality rates (6/14, 43% vs. 18/86, 21%, *P*=0.075).

**TABLE 4:** Statistical Differences Between Free and Contained Rupture Patients

Variable	Overall cases, N (%) median (IQR)	Free rupture N (%) median (IQR)	Contained rupture N (%) median (IQR)	*P* *
Preoperative	**100** (**100)**	**14** (**100)**	**86** (**100)**	
Time to treatment	12 (9)	7 (2-10)	12 (9-23)	0.009
Intraoperative		**14** (**100)**	**86** (**100)**	—
Technical success	89 (89)	8 (57)	81 (94)	<0.001
Operation time (min)	308 (234–453)	446 (260–640)	339 (179–499)	0.006
Postoperative	**98** (**100)**	**13** (**100)**	**85** (**100)**	—
MAEs	33 (34)	9 (69)	24 (28)	0.004
Pulmonary complications	19 (19)	10 (77)	9 (11)	<0.001
30-d reinterventions	24 (24)	8 (62)	16 (19)	<0.001
In-hospital time (d)	13 (8–21)	24 (7–30)	15 (5–13)	0.017
Follow-up time (mo)	13 (5–24)	8 (1–14)	14 (2–25)	0.004

Bold represent the overall number of patients in each subgroup.

Data are presented for continuous data with median and (interquartile range-IQR); categorical data as numbers and (percentage). Data of exclusive statistical significant data are reported in the table together with the *P* value level (significant if <0.05).

* *P* value based on χ^2^ test or with Mann-Whitney *U* test and comparing free ruptured vs. contained ruptured patients.

### Unadjusted and Adjusted Analysis for Primary End Points

At multivariate analysis (Table 5), the presence of contained rupture was the only independent factor favoring technical success [odds ratio (OR): 10.1, 95% CI: 3.0–33.6, *P*<0.001]. For 30-day and/or in-hospital mortality, MAEs (OR: 9.4, 95% CI: 2.8–30.5, *P*<0.001) and pulmonary complications (OR: 11.3, 95% CI: 3.0–41.5, *P*<0.001) were identified as independent risk factors. Regarding follow-up mortality, the presence of an aneurysm diameter >80 mm [hazard ratio (HR: 2.0, 95% CI: 1.0–30.5, *P*=0.037], primary technical failure (HR: 2.6, 95% CI: 1.1–6.5, *P*=0.045), and postoperative pulmonary complications (HR: 3.0, 95% CI: 1.2–7.9, *P*=0.021) were identified as independent risk factors.

**TABLE 5:** Univariate and Multivariate Analysis of Significative Factors for Primary End Points

	Univariate		Multivariate	
Favoring factors for technical success	Unadjusted OR (95% CI)	*P* *	Adjusted OR (95% CI)	*P* †
Contained rupture at presentation	16.8 (0.117–0.671	<0.001	10.1 (3.0–33.6)	<0.001
Risk factors for 30-day+in-hospital mortality				
Technical failure	10.6 (0.077–0.525)	0.004	—	—
ICU>48 h	5.7 (0.081–0.382)	0.017	—	—
MAEs	24.1 (0.306–0.671)	<0.001	9.4 (2.8–30.5)	<0.001
Pulmonary complications	22.5 (0.239–0.676)	<0.001	11.3 (3.0–41.5)	<0.001
Acute kidney injury	4.3 (0.035–0.414)	0.039	—	—
Spinal cord ischemia	5.0 (0.006–457)	0.025	—	—
Bowel ischemia	10.6 (0.016–0.531)	0.002	—	—
30-d reinterventions	4.13 (0.008–0.428)	0.042	—	—
Risk factors for follow-up mortality	Unadjusted		Multivariate	
	HR (95% CI)	*P* *	HR (95% CI)	*P* †
Free rupture at presentation	6.3 (22.7–49.2)	0.012	—	—
Crawford I–III extension	5.3 (13.4–40.5)	0.021	—	—
Aneurysm diameter >80 mm	4.8 (30.1–51.9)	0.028	2.0 (1.0–3-8)	0.037
Technical Failure	23.8 (13.7–49.2)	<0.001	2.6 (1.1–6.5)	0.045
MAEs	18.8 (13.5–40.0)	<0.001	—	—
Pulmonary complications	31.2 (26.2–55.7)	<0.001	3.0 (1.2–7.9)	0.021
Bowel ischemia	7.6 (21.3–50.6)	0.006	—	—
Any reinterventions	4.73 (13.7–40.2)	0.029	—	—

Data of exclusive statistical significant data are reported in the table together with the *P* value level (significant if <0.05).

* *P* value based on χ^2^ test and Log-rank test.

† *P* value based on logistic regression and Cox regression test.

ICU indicates intensive care unit; OR, odd ratio.

## DISCUSSION

This large retrospective study focused exclusively on outcomes of urgent/emergent endovascular repair of rTAAA after exclusion of asymptomatic or symptomatic intact TAAAs. The option of an off-the-shelf multibranched endograft was selected in most patients. The assisted technical success of 93% and early mortality of 24% compare favorably to historical open surgical series of rTAAAs. As expected, outcomes of FB-EVAR for rTAAA are inferior to those reported for intact aneurysms (Supplementary Table S6, Supplemental Digital Content 1, http://links.lww.com/SLA/F13).^21,23–26,35^

Key findings show that factors like the nature (free vs contained) and location (thoracic vs. abdominal) of the aortic rupture, the extension of the aneurysm (type I-III vs type IV), and the patient’s hemodynamic status do not directly influence postoperative and follow-up mortality. As such, these patients should not be immediately written off. Yet, postoperative technical and clinical challenges, especially pulmonary complications, play pivotal roles in patient survival, with a 1-year survival rate at 65%.

The treatment of TAAA is still a major challenge for vascular surgeons: open surgical repairs report early mortality rates of 2.3% to 32.7% for elective^3^ repair, while endovascular repair, especially elective fenestrated and branched endografts (FB-EVAR), reduced mortality as low as 1% in elective cases and reduced morbidities.^9,12,36,37^

The presentation of rTAAA poses a major clinical emergency with definite mortality when left untreated and so far limited literature focuses specifically on rTAAA, with many series combining ruptured aneurysms with symptomatic unruptured and asymptomatic large aneurysms at high rupture risk.^21,23,24,38^ Hongku et al^26^ published a small series exclusively on rTAAA, with 12 patients, whereas Dias-Neto et al^25^ published the widest experience over 2603 F/B-EVAR TAAA treatments out of 24 centers, analyzing both elective and nonelective patients, with only 5.6% of them ruptured (not specified free/contained). A comprehensive larger study specifically on rTAAA was thus needed.

### Endovascular Solutions for rTAAA

In this series of 100 patients, treated within the first 24 hours from diagnosis of rupture, the Cook t-branch off-the-shelf device was predominantly used, showing a primary technical success rate of 88%, confirming its preference as the first choice in cases of urgent/non elective treatment of TAAA, consistent with previous studies.^6,8,11,22,23,25,38^ However, should be noticed that off-the-shelf devices require specific patient’s anatomic features, as indicated by dedicated feasibility studies.^15–17^

Custom-made devices are intriguing options tailored to fit-specific requirements of patients’ anatomies. However, their production and delivery time of ~90 days make them unsuitable for urgent settings.^14^ In cases where off-the-shelf stent grafts cannot be implanted in urgent settings, PMEG have also been proposed:^39,40^ a recent meta-analysis by Gouveia e Melo et al^18^ reported a 95% technical success and an early mortality rate of 10% for urgent patients. In our series, all patients treated with PMEG achieved technical success and survived, although they were all stable patients except one who was stabilized with an emergent TEVAR procedure before undergoing the PMEG. Nevertheless, the application of PMEG requires time and expertise, with techniques and modifications that may vary across different centers. This option could mainly fit Type-I TAAA with narrow aorta at the level of the visceral aorta or in postdissection aneurysms with narrow true lumen.

PGs represent a fourth option^19^ due to the risk of gutter endoleaks and incomplete exclusion of the rupture. In our cases, 2 patients with unfeasible anatomy for off-the-shelf devices and PMEG underwent PG repair, achieving primary technical success.

### End Points of the Study

The primary procedural endpoint of our study was primary technical success, which was achieved in 89 patients and increased to 93 cases after assisted procedures. These findings are consistent with recent literature,^21,23,25^ demonstrating a stable learning curve. Two failures were attributed to intraoperative mortality, highlighting the critical issue of stabilizing the patient during these complex time-consuming procedures.

Regarding early mortality, it occurred in nearly a quarter of the cases, in line with the rates reported by Kölbel et al^21^ of 30% by Gallitto et al^23^ 22% by Dias-Neto et al^25^ of 20% and lower compared with open aortic repair rate of 35%.^35^

Preoperative/anatomic factors were not predictors of early mortality, whereas postoperative conditions and management in resulted independent risk factors. Among MAEs acute kidney injury was the most common, consistent with similar studies.^23,24^ SCI occurred in approximately one fifth of the cases, with 40% of the patients resulting in permanent damage. However, the urgent nature of these cases limited the application of standard protocols for SCI prevention, such as spinal fluid drainage (performed in 30% of patients) or staged techniques.^10,23,24,35,41–43^ At the same time, when planning these complex emergent TAAA endovascular repairs, particular attention was observed in guaranteeing both left subclavian artery^44^ and internal iliac artery perfusion^45,46^ to prevent SCI injuries.^16,47,48^

Pulmonary complications were reported in 19% and were identified as independent factors for early mortality. This finding has been already reported both after open repair (pooled rate of 23%)^3^ and endovascular repair (with ranges from 5% up to 33%).^23–25^ The presence of bleeding in the thoracic compartment may contribute to these complications, highlighting the need for further understanding of the role of preventive thoracic decompression and the risk of subsequent bleeding.

Exclusively during the follow-up period, some preoperative anatomic factors show a role on mortality, which could be speculatively associated with more complex procedures after the first emergency period: I-III Crawford extend TAAA show an increase of mortality in the univariate analysis but not as independent factor mirroring what is reported in similar larger comprehensive experiences;^25^ additionally, preoperative aneurysm diameter >80 mm was associated with mortality even in adjusted analysis, paralleling the definition of “urgent” aneurysms with a diameter >80^23,24^ or 90 mm.^21^ Studies to comprehensively address these issues are needed.

### Free Versus Contained Rupture Analysis

Although our study exclusively included patients with ruptures, we aimed to recognize and highlight the differences between free and contained rupture presentation. So far, no homogeneous definition for “rupture” is reported in the literature;^21,23,26,35,39^ in this study, we tried to provide a clear definition providing consistent results, but further studies are needed to aim to a general consensus.

Specifically, 14 patients presented with free aortic rupture (50% in abdominal and thoracic compartment, respectively), and the location of the aortic rupture did not represent a determining factor. From a technical perspective, the presence of free rupture was associated with lower primary technical success rates and longer procedural times. This finding is consistent with previous literature^24–26,41,49^ and can be attributed to the challenges of performing accurate planning, time-consuming steps required to seal the aneurysm together with the limited availability of dedicated materials in urgent settings and the need for adjunctive maneuvres to maintain patient’s hemodynamic stability throughout the procedure. In case of free ruptures, controlled hypotension was maintained up to endograft deployment; thereafter, mean systemic pressure was augmented to reduce SCI risks due to prolonged hypoperfusion status.

Regarding mortality, both free and contained ruptures had one reported intraoperative death each. In the postoperative phase, patients with free ruptures had twice the mortality rate as those with contained ruptures, although this difference did not reach statistical significance. Postoperatively, complications and the rate of reinterventions were higher in patients with free ruptures.

Therefore, based on these numbers, the presence of a free rupture could not be considered a determinant of mortality itself, suggesting that endovascular intervention is still a viable option for these patients. Nevertheless, caution is advised: patients with free rupture, accompanied by severe hemodynamic instability and loss of consciousness, planned for a complex procedure with low chances of primary technical success or a straightforward postoperative period, should undergo careful evaluation before deciding on repair.

### Limitations

This study has several limitations inherent to its retrospective design and the short follow-up, superimposable to other larger studies,^25^ but uncapable to reach long-term conclusions. Due its retrospective nature, a selection bias is present since exclusive treated cases are reported without data on patients who were not considered for treatment. Similarly, no open procedures were performed for rTAAAs among participating centers (excepting 17 open rTAAA cases in 1 center): results highlight outcomes from institutions with relevant endovascular expertise. The results may be nongeneralizable due to varying expertise in complex endovascular procedures and the availability of specific devices across different centers, resulting in different treatment modalities of the rTAAA cases that should be considered in the technical analysis. At the same time, the limited number of free ruptured patients did not enable us to perform preoperative scoring on this subgroup of patients, and some results might be flawed by statistical type 2 error. Patient inclusion from multiple centers also introduces variations in expertise and management protocols, and the role of surgical thoracic/abdominal decompression techniques should be further analyzed.

## CONCLUSIONS

This study underscores the complexity and risks associated with ruptured thoracoabdominal aortic aneurysms. While endovascular repair, primarily using off-the-shelf multibranched endografts in dedicated centers, permits high technical success, it is notable that the type or location of the rupture either aneurysm extension did not seem to solely dictate survival, thus not serving as a standalone contraindication for the procedure. Careful intraoperative and postoperative care, especially in preventing and rescue from adverse events, especially pulmonary and reinterventions, is pivotal to enhance patient survival.^50^ Expanding research will refine therapeutic approaches and promote specific guidelines.

## Supplementary Material
