# Supplementary material for: Endovascular Repair of 100 Urgent and Emergent Free or Contained Thoracoabdominal Aortic Aneurysms Ruptures. An International Multicenter Trans-Atlantic Experience
Source: Ann Surg. 2024 Feb 7;281(3):522–31. doi: 10.1097/SLA.0000000000006231 (PMC11809711; doi:10.1097/SLA.0000000000006231)
Supplement: Supplementary file 1 [file sla-281-522-s001.docx]

**Manuscript Number: ANNSURG-D-23-01822 (First Revision)**

**SUPPLEMENTAL DATA FILES**

**SUPPLEMENTARY TABLES**

**Table S1: Details of free rupture patients**

| **N** | **Gender, Age(years)** | **Location Rupture SVS** | **Type of Symptoms** | **Blood pressure systolic/diastolic (mmHg)  amine support** | **Aneurysm type Diameter (mm)** | | **Time to treatment (h)** | **Type of treatment (main graft)** | **Need for immediate evacuation** | **Alive or Death (days/months)** |  |
| --- | --- | --- | --- | --- | --- | --- | --- | --- | --- | --- | --- |
| 1 | F, 75 | 4 | Thoracic backpain + left haemothorax | 95/60  Amines support needed | TAAA type II 95 mm | | 4 | T-branch | NO | Alive 23 months |  |
| 2 | M, 73 | 9 | Abdominal pain | 100/65 Amines support needed | TAAA type II 77 mm | | 2 | T-branch | NO | Alive at 13 months |  |
| 3 | M, 62 | 5 | Back pain | 90/40 Amines support needed | TAAA type III 55 mm | | 2 | PMEG | NO | Alive at 5 months |  |
| 4 | M, 69 | 5 | Chest/back pain | 124/80 No need for amines | PAU  (type IV TAAA extension)  34 mm | | 12 | T-branch | NO | Alive at 20 months |  |
| 5 | M, 77 | 5 | Thoracic pain | 110/65  No amines support needed | TAAA type IV 75 mm | | 20 | T-branch | NO | Died after 3 days |  |
| 6 | F, 77 | 4 | Thoracic/chest pain | 120/80  No need for amines | TAAA type IV 140 mm | | 24 | T-branch | YES | Died after 9 months |  |
| 7 | M, 67 | 9 | Abdominal pain | 130/80 No amines support needed | TAAA type IV 80 mm | | 12 | T-branch | NO | Died after 4 months |  |
| 8 | F, 79 | 6 | Chest pain | 80/50 Amines support needed | TAAA type II 57 mm | | 10 | T-branch | NO | Died after 5 days |  |
| 9 | M, 64 | 9 | Abdominal pain | 110/80 No amines support needed | TAAA type II 75 mm | | 6 | T-branch | NO | Intraoperative death |  |
| 10 | F, 85 | 9 | Abdominal pain | 120/80  No amines support needed | TAAA Type IV 85 mm | | 0 | T-branch | NO | Died after 46 days |  |
| 11 | M, 70 | 5 | Chest Pain | 70/20 Amines support needed | TAAA Type III 70 mm | | 1 | T-branch | YES | Died after 24 months |  |
| 12 | M, 78 | 4 | Chest Pain | 105/60 No amines support needed | TAAA type II 95 mm | | 2 | T-branch | YES | Died after 2 days |  |
| 13 | M, 74 | 6 | Abdominal Pain | 117/90 No amines support needed | TAAA type IV 100 mm | | 5 | T-branch | NO | Died after 30 days |  |
| 14 | M, 85 | 8 | Abdominal and back pain | 100/60 No amines support needed | Endoleak IA prior EVAR (type IV TAAA extension) 110 mm | | 2 | T-branch | NO | Alive at 15 months |  |
|  |  |  |  |  |  |  | |  |  | |  |

M=Male; F= Female; SVS = Society for Vascular Surgery; TAAA: Thoraco abdominal aortic aneurysm

**Supplementary Table S2: Details of proximal and distal landing zones (SVS zone).**

| **SVS Zone** | **N** | **Free Rupture N (%)** | **Contained Rupture N (%)** | **T-branch**  **N (%)** | **PMEG**  **N (%)** | **CMD**  **N (%)** | **Parallel Graft**  **N (%)** | **Adjunctive Procedure  N (patients) and details for landing zone** |
| --- | --- | --- | --- | --- | --- | --- | --- | --- |
| **Location of Proximal Landing Zone** | | | | | | | | |
| Zone 1 | 2 | 1 (50) | 1 (50) | 2 (100) | 0 | 0 | 0 | 3 (2)  - (1) Previous arch replacement  - (1) C-C-S bypass + TEVAR |
| Zone 2 | 6 | 2 (33) | 4 (67) | 5 (83) | 0 | 0 | 1 (17) | 12 (6)  - (1) Laser fenestration for LSA + TEVAR  - (4) C-S bypass + TEVAR  - (1) Chimney for LSA + TEVAR |
| Zone 3 | 32 | 10 (31) | 22 (69) | 31 (97) | 1 (3) | 0 | 0 | 35 (32):  - (32) Proximal TEVAR  - (1) Coil Embolization of rupture site  - (1) Embolization of renal branch |
| Zone 4 | 26 | 1 (4) | 25 (96) | 20 (77) | 5 (19) | 0 | 1 (96) | 24 (23)  - (23) proximal TEVAR  - (1) candy PLUG in the false lumen |
| Zone 5 | 34 | 0 | 34 (100) | 30 (88) | 2 (6) | 2 (6) | 0 | - |
| **Location of Distal Landing Zone** | | | | | | | | |
| Zone 9 | 35 | 5 (14) | 30 (86) | 27 (77) | 7 (20) | 0 | 1 (3) | 4 (3)  - CIA lithotripsy and stenting  - stenting of CIA for dissection  - iliac artery angioplasty |
| Zone 10 | 46 | 7 (15) | 39 (85) | 42 (92) | 1 (2) | 2 (4) | 1 (2) | 8 (7)  - CFA angioplasty and iliac paving and cracking  - (4) iliac artery angioplasty  - (2) Iliac – femoral bypass conduit |
| Zone 11 | 19 | 2 (10) | 17 (90) | 19 (100) | 0 | 0 | 0 | 20 (19)  - (15) IBD  - (1) IBD + Contralateral IIA embolization  - (3) cases of iliac rupture and landing zone into external iliac |

Data are presented as numbers (percentage).

SVS; Society for Vascular Surgery; CCS: carotid to carotid subclavian bypass; LSA: Left subclavian artery; CS: carotid subclavian bypass. CMD: Custom Made Devices; PMEG: Physician-Modified Endograft; ; TEVAR: Thoracic Endovascular Repair; CIA: Common iliac artery; CFA: Common femoral artery; IIA: Internal Internal Artery; IBD Iliac Branch Devices;

**Supplementary** **Table S3. Details of technical failures**

| **N** | **Type of Rupture** | **Location Rupture SVS** | **Aneurysm type  Diameter (mm)** | **Time to treatment (h)** | **Type of treatment (main graft)** | **Total operation time (minutes)** | **Reason for Technical Failure** | **Solved (Assisted TS) Details** | **Clinical Evolution** |  |
| --- | --- | --- | --- | --- | --- | --- | --- | --- | --- | --- |
| 1 | Contained | 4 | TAAA type II 72 mm | 2 | T-branch | 235 | Persisting perfusion of false lumen ruptured | YES Coil Embolization of FL | Regular, Alive |  |
| 2 | Contained | 9 | TAAA type IV 78 mm | 4 | T-branch | 186 | Intraoperative death | NO | Intraoperative death |  |
| 3 | Contained | 5 | TAAA type IV 70 mm | 24 | T-branch | 456 | Impossible recanalization of left renal artery | NO Plug embolization of dedicated branch | Death after 13 days for respiratory insufficiency |  |
| 4 | Free | 4 | TAAA type IV 140 mm | 24 | T-branch | 840 | Impossible recanalization of celiac trunk | NO  Plug embolization of dedicated branch | Embolization of left renal artery + thoracotomy Died after 9 months |  |
| 5 | Contained | 9 | TAAA type III 63 mm | 23 | T-branch | 555 | Endoleak type III | YES Endovascular relining | Reinterventions for endoleak and hematoma. Died after 13 months for pneumonia |  |
| 6 | Contained | 5 | TAAA type III  103 mm | 5 | T-branch | 480 | Impossible recanalization of right renal artery | NO Plug embolization of dedicated branch | Renal parenchymal rupture and retroperitoneal hematoma Died after 3 days |  |
| 7 | Free | 9 | TAAA type IV 80 mm | 12 | T-branch | 300 | Parenchymal bleeding from right kidney during the procedure | NO  Emergency right nephrectomy | Temporary dialysis  Death after 4 months |  |
| 8 | Free | 6 | TAAA type II 57 mm | 10 | T-branch | 595 | Endoleak IA during postoperative CTA for instability | NO Hemorrhagic shock | Pulmonary insufficiency due to hemothorax Died after 5 days |  |
| 9 | Free | 9 | TAAA type II 75 mm | 6 | T-branch | 1035 | Intraoperative death | NO | Intraoperative death |  |
| 10 | Free | 4 | TAAA type II 95 mm | 2 | T-branch | 525 | Endoleak type Ib | YES Solved with extra relining | Died after 2 days for myocardial infarct |  |
| 11 | Free | 6 | TAAA type IV 100 mm | 5 | T-branch | 415 | Endoleak type III and Ib + major axillary access bleeding | YES Endovascular relining + axillary artery patching | Respiratory insufficiency due to pneumonia, permanent dialysis, paraplegia  Died after 30 days |  |
|  |  |  |  |  |  |  |  |  | |  |

SVS = Society for Vascular Surgery; TAAA: Thoraco abdominal aortic aneurysm

**Supplementary Table 4: 30-day primary and secondary endpoints**

| **Variable** | **N (%) or Mean (SD)** |
| --- | --- |
| **Overall cases** | **100 (100%)** |
| **Primary endpoints** |  |
| **Technical Success** | **89 (89)** |
| Intraoperative mortality | 2 (2) |
| Other technical failures | 9 (9) |
| **30-day+in-hospital mortality** | **24 (24)** |
| **Clinical secondary endpoints** |  |
| Need for ICU | 82 (84) |
| ICU more than 48 hours | 69 (70) |
| Mean days in ICU | 7 (6) |
| Patients with MAEs | 33 (34) |
| Stroke | 2 |
| Myocardial infarction/failure | 9 |
| Pulmonary complications | 19 |
| Acute Kidney injury | 22 |
| Need for temporary dialysis | 8 |
| Need for permanent dialysis | 4 |
| Permanent SCI (paralysis / paraparesis) | 8 |
| Transient SCI (paralysis / paraparesis) | 12 |
| Bowel ischemia | 5 |
| Acute Limb ischemia | 4 |
| Reinterventions | 24 (24) |
| Clinical Success* | 68 (69) |
| **90-day mortality** | **27 (27)** |

Data are presented as mean (standard deviation-SD) and categorical data as
numbers (percentage). Primary endpoints are calculated on 100 cases. Clinical secondary endpoints percentages are calculated on 98 cases, due to 2 intra-operative mortalities.90-day mortality was calculated on the overall cohort of patients.

ICU: Intensive Care Unit; MAE: Major Adverse Events; SCI: Spinal Cord Ischemia

*: as reported by Reporting Standards^30^ presence of Technical Success and absence of important disabling permanent clinical sequelae.

**Supplementary** **Table S5. Details of 30 day reinterventions**

| **Reason for Reintervention** | **N (%)** | **Free rupture N (%)** | **Contained Rupture N(%)** | **Type of rupture and complication** | **Type of reintervention** | **Clinical Evolution** |
| --- | --- | --- | --- | --- | --- | --- |
| Access related | 7 (30) | 2 (29) | 5 (71) | Contained Rupture; Occlusion of right femoral artery | 1. Surgical Thrombectomy and patching - Solved | Alive |
|  |  |  |  | Contained Rupture; Dissection of iliac artery | 1. Iliac artery relining via bare metal stent- Solved | Died  (10 days) |
|  |  |  |  | Contained Rupture; pseudoaneurysm of right groin | 1. Surgical repair - Solved | Alive |
|  |  |  |  | Contained Rupture; Dissection of right iliac artery | 1. Endovascular relining - Solved | Alive |
|  |  |  |  | Contained Rupture; Axillary artery bleeding | 1. Surgical revision - Solved | Alive |
|  |  |  |  | Free Rupture; Groin access dehiscence | 1. Multiple surgical revisions - Failure | Died  (46 days) |
|  |  |  |  | Free Rupture; Multiple Bleeding from axillary access and retroperitoneal bleeding | 1. Multiple surgical revisions - Solved | Died  (24 months) |
| TVV relining | 3 (12) | 1 (33) | 2 (67) | Free Rupture; Endoleak type IIIc from CT | 1. Endovascular Relining with stentgraft - Solved | Alive |
|  |  |  |  | Contained Rupture; Endoleak type Ic from CT branch | Endovascular Relining with stentgraft - Solved | Died  (5 days) |
|  |  |  |  | Contained Rupture; Endoleak type Icfrom SMA branch | Endovascular relining with stentgraft - Solved | Alive |
| Main endograft relining | 4 (17) | 2 (50) | 2 (50) | Contained Rupture; Persistent of flow in false lumen (type III) | TEVAR relining - Solved | Alive |
|  |  |  |  | Contained Rupture; Type 1B endoleak | Endovascular relining with IIA occlusion - Solved | Alive |
|  |  |  |  | Free Rupture; Endoleak1A proximal and new hemodynamic shock | Additional relining proximal TEVAR - Unsolved | Died  (5 days) |
|  |  |  |  | Free Rupture; Type III endoleak between thoracic and T-branch modules | Relining with extra-TEVAR module - Solved | Died  (30 days) |
| Active bleeding | 4 (17) | 2 (50) | 2 (50) | Contained Rupture; retroperitoneal bleeding | Surgical hemostasis and evacuation- Unsolved | Died (36 days) |
|  |  |  |  | Contained Rupture; Bleeding from artero-venous fistula in iliac limb | Endovascular relining with extra iliac limb - Solved | Alive |
|  |  |  |  | Free Rupture; Left kidney parenchymal bleeding | Embolization of left renal artery - Solved | Died  (9 months) |
|  |  |  |  | Free Rupture; Right kidney parenchymal bleeding | Hemostasis evacuation and subsequent right kidney nefrectomy - Unsolved | Died (4 months) |
| Unplanned hematoma evacuations | 3 (12) | 1 (33) | 2 (67) | Free Rupture; Hemothorax with lung atelectasis | Post-operative thoracoscopic evacuation - Solved | Alive |
|  |  |  |  | Contained Rupture;  Persistent pleural effusion | Surgical thoracotomy - Solved | Alive |
|  |  |  |  | Contained Rupture; Retroperitoneal hematoma | Evacuation of retroperitoneal abdominal hematoma- Solved | Died  (13 months) |
| Bowel ischemia | 2 (8) | 0 | 2 (100) | Contained Rupture;  Mesenteric Ischemia | Resection of ileum - Unsolved | Died  (18 days) |
|  |  |  |  | Contained Rupture;  Mesenteric Ischemia | Left hemicolectomy - Solved | Died  (56 days) |
| Graft thrombosis | 1 (4) | 0 | 1 (100) | Contained Rupture; Right iliac limb occlusion | Right femoral access thrombectomy and stentgrafft relining | Died  (27 days) |
| **OVERALL** | **24 (100)** | **8 (33)** | **16 (67)** |  |  |  |

**Supplementary Table S6: Summary of main outcomes with recent Literature**

| **Author (year)**  **Journal** | **Overall**  **patients**  **N** | **Symptomatic cases**  **N (%)** | **Ruptured Cases**  **N (%)** | **Type of repair***  **N (%) details** | **Type of main endograft***  **N + details** | **TechnicSucces***  **%** | **30-day or**  **In-hospital mortality***  **%** | **SCI***  **%** | **Pulmonary complication***  **%** | **New onset dialysis***  **%** | **30-day redo***  **%** | **1-year survival***  **%** |
| --- | --- | --- | --- | --- | --- | --- | --- | --- | --- | --- | --- | --- |
| **Spath (2023)** | **100** | **100  (100)** | **100 (100)** | **100 (100)**  **Endovascular** | **88 t-branch 8 PMEG**  **2 PG  2 CMD** | **89** | **24** | **20** | **19** | **12** | **24** | **65** |
| **Dias-Neto (2023) Ann Surg**^25^ | 2603 | 268 (16) | 148 (5.6) | 148 (100) Endovascular | 133 t-branch  15 PMEG | 95 | 20 | 17 | 14 | 22** | - | 62 |
| **Gallitto**^23^ **(2022) EJCTS** | 65 | 35  (54) | 27 (42) | 27 (100) Endovascular | 27 t-branch | 93 | 22 | 30 | 33 | 15 | 33 | 56 |
| **Eleshra**^24^ **(2022) JVS** | 100 | 70 (70) | 21 (21) | 100 (100) Endovascular | 21 t-branch | 95 | 24 | 38 | 5 | 19 | - | 87 |
| **Kolbel**^21^ **(2021)**  **JVS** | 542 | 213 (37) | 46 (9) | 46 (100) Endovascular | 46 t-branch | 97 | 30 | - | - | - | - | - |
| **Hongku**^26^ **(2018)**  **EJVES** | 12 | 12 (100) | 12 (100) | 12 (100)  Endovascular | 11 t-branch  1 CMD | 64 | 27 | 27 | - | 33 | 8 | 75 |
| **Gombert**^35^ **(2022)**  **EJVES** | 255 | 51 (20) | 51 (20) | 51 (100)  Open Surgical Repair | - | - | 35 | 12 | 45 | 35 | - | 55 |

* cases are calculated on ruptured patients; PMEG: Physician Modified Endograft; CMD; Custom Made Devices; PG: Parallel Graft.

** not reported if permanent/temporary dyalisis

**SUPPLEMENTARY FIGURE**

**Supplementary Figure S1**

**
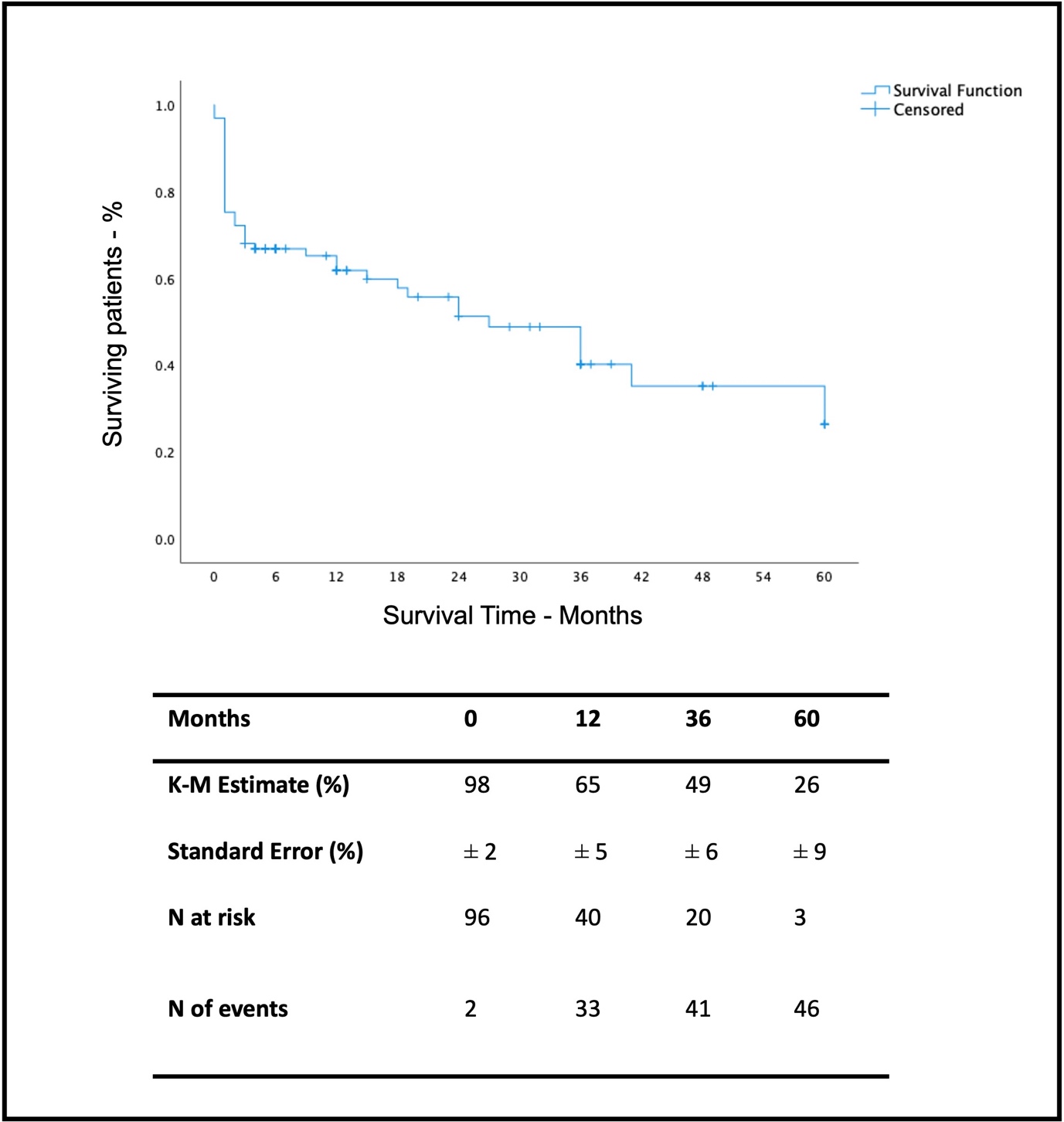
**

**Supplementary Figure S1: Kaplan Meier’s Survival Analysis.**  Overall survival through follow-up time. In the table are reported estimates and standard errors (percentages-%) and numbers (N) of patients at risk and events at 1, 12, 36 and 60 months follow-up. The two intra-operative mortalities were excluded from analysis.

**Supplementary Figure S2**

**
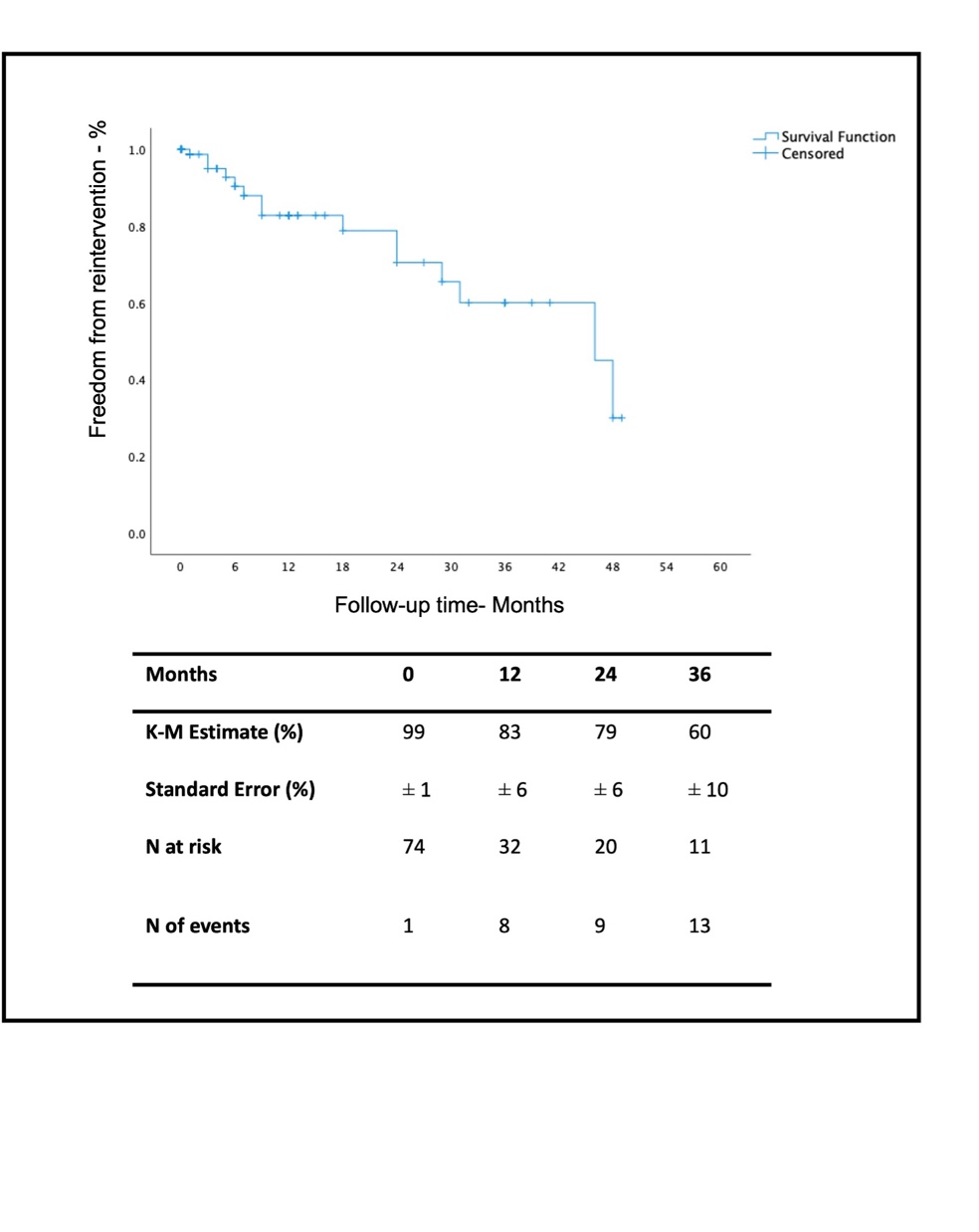
**

**SUPPLEMENTARY FIGURE LEGENDS**

**Supplementary Figure S2: Kaplan Meier’s Freedom from reinterventions Analysis.**  Overall freedom from reintervention through follow-up time. In the table are reported estimates and standard errors (percentages-%) and numbers (N) of patients at risk and events at 0, 12, 36 and 60 months follow-up.
